# Association between *NINJ2* gene polymorphisms and ischemic stroke: a family-based case–control study

**DOI:** 10.1007/s11239-014-1065-6

**Published:** 2014-03-25

**Authors:** Yanping Zhu, Kuo Liu, Xun Tang, Jinwei Wang, Zhiping Yu, Yiqun Wu, Dafang Chen, Xueyin Wang, Kai Fang, Na Li, Shaoping Huang, Yonghua Hu

**Affiliations:** 1 Department of Epidemiology and Biostatistics, Peking University Health Science Center, 38 Xueyuan Road, Beijing, 100191 China; 2 Department of Nephrology, Peking University First Hospital, Beijing, China; 3 Fangshan District Bureau of Health, Beijing, China

**Keywords:** Ischemic stroke, *NINJ2* gene, Family-based study, Association, Interaction

## Abstract

Novel susceptibility genes related to ischemic stroke (IS) are proposed in recent literatures. Population-based replicate studies would cause false positive results due to population stratification. 229 recruit IS patients and their 229 non-IS siblings were used in this study to avoid population stratification. The family-based study was conducted in Beijing from June 2005 to June 2012. Association between SNPs and IS was found in the sibship discordant tests, and the conditional logistic regression was performed to identify effect size and explore gene–environment interactions. Significant allelic association was identified between *NINJ2* gene rs11833579 (*P* = 0.008), *protein kinase C η* gene rs2230501 (*P* = 0.039) and IS. The AA genotype of rs11833579 increased 1.51-fold risk (95 % CI 1.04–3.46; *P* = 0.043) of IS, and it conferred susceptibility to IS only in a dominant model (OR 2.69; 95 % CI 1.06–6.78; *P* = 0.036]. Risk of IS was higher (HR 3.58; 95 % CI 1.54–8.31; *P* = 0.003) especially when the carriers of rs11833579 AA genotype were smokers. The present study suggests A allele of rs11833579 may play a role in mediating susceptibility to IS and it may increase the risk of IS together with smoking.

## Introduction

Stroke is the leading cause of neurological disability and it is one of the leading causes of death worldwide [1], and ischemic stroke (IS) is the major type of stroke in China [2]. Although genome wide association study (GWAS) and genetic studies based on candidate genes have provided numerous probable susceptibility locus, a large part of genetic risks of IS remains unexplained.

Several candidate genes were identified for IS based on genome-wide linkage search and subsequent fine-mapping in the past decades, such as *PDE4D* gene [3], *ALOX5AP* gene [4], and *protein kinase C η* (*PRKCH*) gene [5]. Besides, candidate genes were also proposed and demonstrated based on different pathways of pathogenic mechanisms, such as *Selenoprotein S* (*SEPS1*) gene which involved in the regulation of inflammatory response and protection from oxidative damage [6]. GWAS has been used in exploring genetic risk factors of IS since 2007, and has provided more replicable novel genetic markers, such as rs10757278 in 9p21 [7] and rs11833579 encoded NINJ2 [8]. Numerous replicate studies are needed to perform in various populations in order to provide more solid evidence of causality. To date, most studies aim at confirming previous susceptibility locus are population-based case control study, however, this kind of study design will cause misleading false positive results because of population stratification. Therefore, we used a random non-IS sibling of IS patients as matched control, and carried out family-based case control study to confirm the association between single nucleotide polymorphisms (SNPs) in several novel genes and IS.

As with most complex diseases, IS is likely to be influenced not only by genetic, but also by environmental components. Traditional cardiovascular disease risk factors, such as cigarette smoking and alcohol drinking, may increase the risk of IS [9, 10]. Some of these traditional risk factors may dramatically increase risk of disease together with risk alleles. Identification of interactions between genetic and environment components will help to recognize individuals with high risk in developing IS, and make cost-effective prevention strategies. Thus, the joint effects of traditional environment risk factors and genes which have main effect in IS were also investigated in the present study.

## Methods

### Study population and data collection

Following the protocol of Fangshan/Family-based Ischemic Stroke Study in China (FISSIC) [11], the subjects were recruited in Fangshan from June 2005 to June 2012. Fangshan located in southwest of Beijing is a district with high prevalence of cardiovascular disease including IS [12]. IS patients aged above 18 years old with computerized tomography (CT) or magnetic resonance imaging (MRI) confirmed were recruited in the First Hospital of Fangshan District. Patients are eligible for inclusion if they have at least one parents or a full sibling aged above 18 years participating in the study.

Demographic characters (age, sex, etc.), lifestyle risk factors (cigarette smoking etc.) and medical history (diagnose and treatment of stroke and other chronic disease) were collected through a face-to-face interview by trained interviewers using uniform questionnaires. Current smoker was defined as the one who smoked more than 100 cigarettes and still smoking during last 1 month, past smoker was defined as the one who had smoked that amount of cigarettes but have not smoke for at least 1 month, and non-smoker are those who has smoked <100 cigarettes accumulatively. In the analysis, current smokers and past smokers were both treated as smokers. Anthropometric measurements were obtained by trained and certified observers. Blood pressure was measured three times at the right brachial artery using a sphygmomanometer after the participant has rested in a seated position for 5 min. Concentrations of total cholesterol, triglycerides, and high-density lipoprotein cholesterol were measured with Hitachi 7180 auto-analyzer (Hitachi High-Technologies Corp., Tokyo, Japan) in central clinical laboratory, Central Hospital.

This study had been approved by the ethics committee of Peking University Health Sciences Center. Each participant had been informed about the study and they had agreed for blood sampling and physical examination in this study. All subjects had signed an informed consent prior to participating in the study. In the end, we recruited 229 unrelated Chinese patients with their 229 full sibling non-IS controls.

### DNA extraction and genotyping

We selected 13 SNPs to test their association with IS. Genomic DNA was extracted from the peripheral blood with a DNA extraction kit (DP319-01; Tiangen Biotech, Beijing, China). The PCR was performed on a thermal cycler (ABI GeneAmp 9700 384 Dual; ABI, Foster City, CA, USA). Genotypes were analyzed with Typer 4.0 software (MassARRAY Compact System; Sequenom, San Diego, CA, USA). All SNPs in this report had a genotyping success rate of >98 %.

### Statistical analysis

The initial data set contains singleton families and multiplex families. The 199 singleton families contain only one affected individual and at least one unaffected siblings. The 44 multiplex families comprise families with at least 2 affected siblings, and any number of unaffected siblings. After excluding 14 multiplex families which have no unaffected siblings, we recruited a total of 229 families in the association study. For this study, in affected group, one case from each family was selected. In unaffected group, one non-IS siblings of IS patient from each families was randomly selected.

During data analysis process, means and standard deviations (SDs) were reported for continuous variables, and percentages were reported for categorical variables. Paired sample *t* test was used to examine the difference of continuous variables, and McNemar’s Chi squared tests were used to test for the difference of categorical variables. Each SNP was tested separately for deviation from Hardy–Weinberg equilibrium (HWE) in control group using Chi squared test. We used the sibship disequilibrium test (SDT) to test association between SNPs and IS in the first step. Conditional logistic regression served as a second method to confirm the SDT results and provided estimates of relative risks. We analyzed the family-based data, by using the procedure COX regression in IBM SPSS Statistics 20.0 software, to fit the conditional logistic regression model and search for gene-environment interactions. All *P* values were two-tailed, with statistical significance defined as <0.05.

## Results

We used 229 case-sibling pairs in this association study. Of the 229 IS patients, 157 (57.9 %) were male, which were significantly more than control group (*P* < 0.001, Table 1). The patients were significantly elder than controls (*P* = 0.002). The percent of IS cases that had ever smoked cigarettes was significantly higher than controls (58.6 vs. 41.4 %, *P* = 0.001). Systolic and diastolic blood pressures were significantly higher in cases than controls (*P* ≤ 0.001). There was no significant difference of the concentration of total cholesterol between patients and controls (*P* = 0.366), while the concentration of HDL-cholesterol (HDLC) was significantly higher in patients than controls (*P* < 0.001).

**Table 1:** Clinical characteristics of cases and their unaffected siblings

	Affected	Unaffected	*t*/χ^2^	*P* value
Age (years)	61.08 ± 10.885	58.63 ± 9.13	3.103	0.002**
Male	157 (57.9 %)	114 (42.1 %)	16.711	<0.001***
Smoke	139 (58.6 %)	98 (41.4 %)	14.896	0.001**
Diabetes	61 (67.0 %)	30 (33.0 %)	13.719	<0.001***
Waist (cm)	92.16 ± 8.850	88.16 ± 9.536	4.899	<0.001***
BMI (kg/m^2^)	26.86 ± 3.987	26.05 ± 3.598	2.435	0.016*
Hypertension	173 (61.3 %)	109 (38.7 %)	37.798	<0.001***
Systolic blood pressure (mmHg)	144.72 ± 18.344	134.21 ± 19.921	6.172	<0.001***
Diastolic blood pressure (mmHg)	90 ± 10.942	86.21 ± 11.059	3.432	0.001**
Dyslipidemia	166 (54.2 %)	140 (45.8 %)	6.657	0.010*
TC (mmol/l)	4.70 ± 1.059	4.63 ± 1.091	0.907	0.366
HDLC (mmol/l)	1.42 ± 0.43	1.57 ± 0.507	−4.400	<0.001***

*BMI* body mass index, *SBP* systolic blood pressure, *DBP* diastolic blood pressure, *TC* total cholesterol, *HDLC* high density lipoprotein cholesterol

* *P* < 0.05; ** *P* < 0.01; *** *P* < 0.001

All 13 SNPs showed no significant deviation from Hardy–Weinberg equilibrium in the control group (*P* > 0.05). The results of SDT and allele frequencies for patients and controls were shown in Table 2. Significant associations with IS were found in *NINJ2* gene rs11833579 (*P* = 0.008), *PRKCH* gene rs2230501 (*P* = 0.039). The A allele frequency in rs11833579 was significantly higher in stroke patients (40.0 %) than that of unaffected controls (33.6 %). The C allele of rs2230501 is significantly higher in stroke patients (22.9 %) than that of unaffected controls (19.4 %).

**Table 2:** SDT results and allele frequency for 14 SNPs

	Gene/location	Allele	Affected	%	Unaffected	%	*P* value	HWE *P* value
rs266729	*ADIPOQ*	C	343	72.06	351	75.65	0.144	0.164
		G	133	27.94	113	24.35		
rs520540	*MMP3*	G	293	61.55	290	62.5	0.622	0.302
		A	183	38.45	174	37.5		
rs966221	*PDE4D*	T	360	76.27	357	77.27	0.745	0.319
		C	112	23.73	105	22.73		
rs1800796	*ALOX5AP*	C	307	64.5	311	67.03	0.103	0.144
		G	169	35.5	153	32.97		
rs2200733	Intergenic	T	245	51.91	238	51.52	0.967	0.297
		C	227	48.09	224	48.48		
rs2230501	*PRKCH*	A	367	77.1	374	80.6	0.039*	0.654
		C	109	22.9	90	19.4		
rs7178239	*SELS*	G	242	50.84	240	51.72	0.622	0.215
		C	234	49.16	224	48.28		
rs7193343	*ZFHX3*	T	324	68.07	315	67.89	0.596	0.351
		C	152	31.93	149	32.11		
rs9536591	Intergenic	C	289	60.71	299	64.44	0.051	0.931
		A	187	39.29	165	35.56		
rs10204475	*ZNF650*	G	409	85.92	392	84.85	0.998	0.971
		T	67	14.08	70	15.15		
rs107572778	Intergenic	A	247	55.13	227	52.55	0.835	0.136
		G	201	44.87	205	47.45		
rs11052413	Intergenic	G	421	90.73	413	89.78	0.746	0.089
		T	43	9.27	47	10.22		
rs11833579	*NINJ2*	G	282	60	304	66.38	0.008**	0.390
		A	188	40	154	33.62		

* *P* < 0.05; ** *P* < 0.01

Conditional logistic regression validated the significant association of rs11833579 and rs2230501 with IS. Table 3 showed that, before any adjustments, the A allele of rs11833579 was the risk allele of IS in dominant model [odds ratio (OR) 2.17, 95 % CI 1.01–4.76, *P* = 0.049]. The AA genotype of rs11833579 increased risk of IS 3.04-fold relative to the GG genotype (95 % CI 1.14–8.16, *P* = 0.027). After adjusting for age, sex, hypertension, diabetes, serum lipids and smoke, the A allele of rs11833579 could increase 2.69-fold risk of IS (95 % CI 1.06–6.78; *P* = 0.036) in dominant model. The AA genotype of rs11833579 could increase 1.51-fold risk of IS (95 % CI 1.04–3.46, *P* = 0.043). For rs2230501, after multiple adjustments, the AC genotype gave a 2.93-fold increased risk of IS relative to the AA genotype (95 % CI 1.07–8.00, *P* = 0.036).

**Table 3:** Conditional logistic regression results of rs11833579 and rs2230501

	Variable	Unadjusted				Adjusted^a^			
		Hazard ratio	Lower 95 % CI	Upper 95 % CI	*P* value	Hazard ratio	Lower 95 % CI	Upper 95 % CI	*P* value
rs11833579	GG(ref)	–	–	–	–	–	–	–	–
	AG	1.109	0.766	2.591	0.270	0.987	0.435	2.237	0.975
	AA	3.047	1.138	8.162	0.027*	1.506	1.039	3.458	0.043*
	Recessive	1.567	0.904	2.716	0.110	1.161	0.588	2.290	0.688
	Dominant	2.171	1.011	4.755	0.049*	2.687	1.064	6.783	0.036*
rs2230501	AA(ref)	–	–	–	–	–	–	–	–
	AC	1.639	0.811	3.311	0.169	2.930	1.073	7.996	0.036^§^
	CC	1.794	0.413	7.783	0.435	1.210	0.171	8.564	0.849
	Recessive	1.869	0.829	4.213	0.132	1.869	0.829	4.213	0.132
	Dominant	1.431	0.435	4.707	0.555	1.031	0.237	4.493	0.967

* Compared with GG genotype, *P* < 0.05

^§^Compared with AA genotype, *P* < 0.05

^a^Adjusted for age, sex, hypertension, diabetes, TC, HDLC, smoke

Smoking had significant interaction with rs111833579 in IS (Table 4). The odds ratios (OR) to IS rise continuously as the increasing number of risk allele in rs11833579 and ORs rise more distinct if risk allele carriers were also smokers. The AA genotype could increase 3.58 times of risk in smokers than GG genotype in non-smokers (95 % CI 1.54–8.31, *P* = 0.003), and 3.17-fold IS risk than GG/GA genotype non-smokers (95 % CI 1.46–6.88, *P* = 0.004). After adjusting for age and sex, the AA genotype of smokers could increase 2.72-fold risk of IS (95 % CI 1.09–6.78, *P* = 0.032) than the GG genotype of non-smokers, and increase 2.41-fold risk of IS than GG/GA genotype non-smokers (95 % CI 1.04–5.61, *P* = 0.041).

**Table 4:** Interaction of rs11833579 in *NINJ2* gene with smoke in ischemic stroke

	Unadjusted				Adjusted^a^			
	OR	Lower 95 % CI	Upper 95 % CI	*P* value	OR	Lower 95 % CI	Upper 95 % CI	*P* value
Smoke								
No								
GG(ref)	–	–	–	–	–	–	–	–
GA	1.246	0.689	2.252	0.467	1.219	0.674	2.208	0.512
AA	1.852	0.752	4.559	0.180	1.781	0.696	4.563	0.229
Yes								
GG	2.085	1.138	3.819	0.017*	1.528	0.754	3.099	0.240
GA	2.394	1.332	4.297	0.003**	1.636	0.820	3.264	0.162
AA	3.575	1.537	8.314	0.003**	2.716	1.087	6.780	0.032*
Smoke								
No								
GG(ref)				–				–
GA + AA	1.349	0.766	2.373	0.300	1.315	0.746	2.319	0.343
Yes								
GG	2.085	1.138	3.819	0.017*	1.542	0.763	3.114	0.227
GA + AA	2.625	1.505	4.581	0.001**	1.859	0.962	3.597	0.065
Smoke								
No								
GG + GA(ref)				–				–
AA	1.639	0.710	3.789	0.247	1.597	0.659	3.865	0.300
Yes								
GG + GA	1.988	1.324	2.986	0.001**	1.408	0.829	2.392	0.206
AA	3.165	1.456	6.876	0.004**	2.411	1.037	5.613	0.041*

* *P* < 0.05; ** *P* < 0.01

^a^Adjusted for age and sex

## Discussion

Our study described the association between novel SNPs and IS using case-sibling matched case–control study in Chinese population. We revealed that rs11833579 near *NINJ2* gene was significantly associated with IS. In addition, we also found a significant interaction of rs11833579 and smoking in IS. The strength of our study include well characteristic IS patients with imaging data, and the usage of non-IS sibling as matched control, so as to reduce false positive rate due to population stratification. Additionally, Fangshan District, where our study was conducted, was located in the “stroke belt” of China similar to the “stroke belt” in the south-eastern United States, and its population was representative of the rural northern Han Chinese. Besides, to our knowledge, it is the first study to examine the interaction of smoke and rs11833579 in IS.

Ninjurin2 (NINJ2), which is a cell surface adhesion molecule, plays a role in nerve regeneration via hemophilic interaction [13]. Rs11833579 locates on chromosome 12p13 proximity to *NINJ2* gene and encode Ninjurin2. The result of our study was consistent with the previous genome-wide association study which demonstrated that rs11833579 were associated with IS in Caucasians [8]. Similarly, some replicate study in Asia also found the association between rs11833579 and IS [14, 15]. Tong et al. [15] reported that the A allele of rs11833579 was significantly associated with IS in the Han population (OR 1.27, 95 % CI 1.08–1.49), and rs11833579 conferred susceptibility to IS in recessive model (*P* = 0.004). On the contrary, another Chinese study showed that there was no statistically significant association between rs11833579 and IS [16]. Besides, a study included 137 IS patients and 66 controls from Nanjing China, also found no association between rs11833579 and IS [17]. The difference may be explained by the several possibilities: (1) Our study was conducted in a population with stable genetic background in North China, the studies provide different results recruited their samples in South China [16, 17], the different genetic background may confers to the difference; (2) The living habits are different between North and South of China, since IS is a complex disease affected by both genetic and environment, the complex interaction of different environmental risk factors with SNPs may explain the difference; (3) Some population based case–control studies with limited sample size may lack of power to detect associations with alleles with a small effect. Although the results remain controversial, our results provide additionally evidence for the association between rs11833579 and IS with relatively lower false positive rate.

In addition, our study demonstrated that rs2230501 in *PRKCH* gene had significant association with IS. Protein kinase C mediates a wide variety of signaling pathways [18] and involves in the induction of inducible nitric oxide synthase (iNOS) which lead to vascular endothelial dysfunction [19]. Moreover, it also plays an important role in the development of atherosclerotic diseases [5]. To our knowledge, this is the first study to replicate the association between rs2230501 and IS. Another SNP rs2230500, located near and almost in absolute linkage disequilibrium (LD) with rs2230501 (D′ = 0.99), is the most frequently studied SNP in *PRKCH* gene. A large-scale genetic epidemiological study [5] in Japanese subjects first showed that a nonsynonymous SNP rs2230500 in the *PRKCH* gene with cerebral infarction. Chinese researchers also demonstrated that rs2230500 was significantly associated with IS (OR 1.31; 95 % CI 1.08–1.60; *P* = 0.0058) in a dominant model. After age and sex adjustment, the associations remained significant (OR 1.37, 95 % CI 1.12–1.67, *P* = 0.0019) [20]. Our study showed the heterozygote of rs2230501, which is in absolute LD with rs2230500, could increase the risk of IS compared with wild homozygote. More studies are still need to confirm this association with larger sample size and higher statistical power in Chinese Han population.

In addition, our study found that smoking could increase the risk of IS together with rs11833579. It is known that smoking affects cell-mediated immune responses, causes oxidative damage and arterial endothelial dysfunction [21, 22]. Ninjurin2 is a hemophilic adhesion molecule, which is nerve injury-induced and expressed in many tissues including peripheral nerves [13]. Although the independent role of smoke and *NINJ2* gene is relatively clear, the mechanism involved their joint effect on IS is unknown. It is possible that NINJ2 affects IS by influencing the reaction to brain cells to injury, which might be modified by the oxidative damage or inflammation due to smoke. More replicable studies based on large population are needed to confirm this result and reveal the underlying mechanism of the interaction.

There are several limitations in our study. First, the sample size is relatively small, since it is difficult to recruit IS patients together with their discordant siblings. One of the largest family-based IS studies in the western countries is siblings with ischemic stroke study (SWISS), which recruited 223 probands and 84 stroke-unaffected siblings. Although our sample size is not as large as some population-based case control studies, it is larger than most IS family-based studies. Second, associations of SNPs with subtypes of IS were not examined in the present investigation due to the limited sample size. Thus, studies are needed to examine the association of *NINJ2* gene variation in different IS subtypes in the future so as to provide further evidence for the pathogenic mechanisms of IS.

In conclusion, we confirmed that *NINJ2* genetic variants were related to risk of IS in the northern Chinese population, and found the interaction between rs11833579 and smoking. Further research is needed to confirm this interaction so that the pathogenic mechanisms of IS can be featured and intervened.
